# Interplay between Hepatitis E Virus and Host Cell Pattern Recognition Receptors

**DOI:** 10.3390/ijms22179259

**Published:** 2021-08-26

**Authors:** Pradip Devhare, Mridula Madiyal, Chiranjay Mukhopadhyay, Shiran Shetty, Shamee Shastry

**Affiliations:** 1 Manipal Institute of Virology, Manipal Academy of Higher Education, Manipal 576104, Karnataka, India; chiranjay.m@manipal.edu; 2 Department of Microbiology, Kasturba Medical College, Manipal Academy of Higher Education, Manipal 576104, Karnataka, India; mridula.m@manipal.edu; 3 Department of Gastroenterology and Hepatology, Kasturba Medical College, Manipal Academy of Higher Education, Manipal 576104, Karnataka, India; shiran.shetty@manipal.edu; 4 Department of Immunohaematology and Blood Transfusion, Kasturba Medical College, Manipal Academy of Higher Education, Manipal 576104, Karnataka, India; shamee.girish@manipal.edu

**Keywords:** hepatitis E virus, pattern recognition receptors, interferon, interferon-stimulated genes, innate immune response, inflammation

## Abstract

Hepatitis E virus (HEV) usually causes self-limiting acute hepatitis, but the disease can become chronic in immunocompromised individuals. HEV infection in pregnant women is reported to cause up to 30% mortality, especially in the third trimester. Additionally, extrahepatic manifestations like neuronal and renal diseases and pancreatitis are also reported during the course of HEV infection. The mechanism of HEV pathogenesis remains poorly understood. Innate immunity is the first line of defense triggered within minutes to hours after the first pathogenic insult. Growing evidence based on reverse genetics systems, in vitro cell culture models, and representative studies in animal models including non-human primates, has implicated the role of the host’s innate immune response during HEV infection. HEV persists in presence of interferons (IFNs) plausibly by evading cellular antiviral defense. This review summarizes our current understanding of recognizing HEV-associated molecular patterns by host cell Pattern Recognition Receptors (PRRs) in eliciting innate immune response during HEV infection as well as mechanisms of virus-mediated immune evasion.

## 1. Introduction

Hepatitis E virus (HEV) is one of the major causes of acute, self-limiting viral hepatitis (AVH) which is now recognized as a global health problem in both developing and industrialized regions [1,2,3]. According to WHO estimates in 2015, annually 20 million HEV infections cause 3.3 million symptomatic cases and account for 3.3% of the mortality due to viral hepatitis [4]. The feco-oral route transmission of HEV through contaminated drinking water is attributed to large-scale epidemics in developing countries while zoonotic transmission through the consumption of undercooked meat, blood transfusions, and organ transplantation are considered as major sources of HEV infections in developed nations [3]. Though HEV infection causes self-limiting disease in most young adults, pregnant women from developing countries are at high risk accounting for ~30% of the fatality rate in the third trimester [5,6]. The mechanisms underlying HEV pathogenesis during pregnancy are undermined and related mostly to hormonal imbalance and immunological alterations [5,6]. Chronic hepatitis E has been reported in immunocompromised patients such as organ transplant recipients, HIV-infected individuals, and patients with hematological malignancies (reviewed in [7]). Moreover, extrahepatic manifestations like neuronal and renal diseases and pancreatitis are also reported during the course of HEV infection [8,9].

HEV is a member of the family *Hepeviridae* which is classified into two genera: *Orthohepevirus* and *Piscihepevirus* [10]. *Orthohepevirus* comprises four species (A–D) whereas the *Piscihepevirus* genus includes only the Cutthroat trout virus. Eight different genotypes (HEV 1—HEV 8) have been identified in *Orthohepevirus A* out of which genotypes 1/2/3/4/7 are known to be pathogenic in humans. HEV genotype 3, 4, and 7 are zoonotic viruses. Other species of HEV that infect animals but are not transmissible to humans are *Orthohepevirus B* (chicken), *Orthohepevirus C* (rat, ferret), *Orthohepevirus D* (bat) (Table 1) [10,11]. Among the eight genotypes classified in *Orthohepevirus A* species, genotype 1 and 2 are found to be more virulent during pregnancy and responsible for an increased risk of miscarriage, preterm delivery, and stillbirth. The most prominent genotype (genotype 3) from industrialized countries has been exclusively reported in chronic hepatitis E cases [5,6,7].

**Figure 1:**
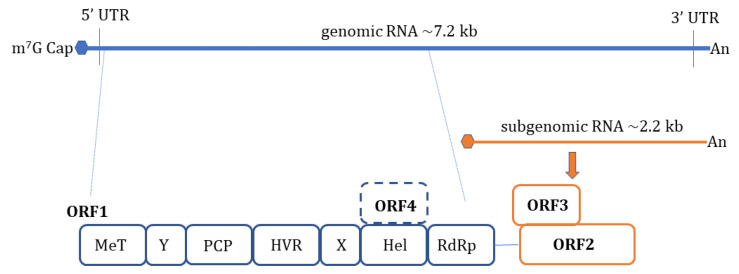
Schematic illustration of Hepatitis E virus (HEV) genome organization. The HEV genome is a single-stranded positive-sense RNA of ~7.2 kb in length. It has a 7- methylguanosine (m7G) cap at the 5′ end and a poly-A tail at the 3′ end. The coding region is flanked by 5′- and 3′- UTRs (Untranslated regions), respectively. The largest open reading frame, ORF1, has several predicted domains, including methyltransferase (MeT), Y domain (Y), papain-like cysteine protease (PCP), hypervariable region (HVR), X domain (X), helicase (Hel), and RNA-dependent RNA polymerase (RdRp). ORF2 and ORF3 are encoded by a subgenomic RNA of ~2.2 kb. ORF2 is the capsid protein. ORF3 partially overlaps with ORF2. The novel ORF4 which is overlapped with ORF1 is only identified in genotype 1.

HEV is a non-enveloped, spherical particle with a diameter of 27–34 nm when it is isolated from bile and feces whereas it also exists as a quasi-enveloped virus (~40 nm) in blood and cell culture supernatants [3]. The single-stranded, positive-sense RNA genome of ~7.2 Kb encompasses three open reading frames (ORFs): ORF1, ORF2, and ORF3 which are flanked by short untranslated regions (UTRs) at 5′ and 3′-ends. The 5′-end is m7G-capped (7-methylguanosine) and the 3′- end is polyadenylated. Recently, an additional ORF (ORF4) has been described to be expressed by genotype 1 HEV which aids in efficient virus replication under stress conditions [12] (Figure 1). Yadav et al. (2021) demonstrated that the ectopic expression of genotype 1 HEV ORF4 enhanced replication of genotype 3 which does not code for ORF4 [13]. Nonstructural proteins such as methyltransferase (MT), a papain-like cysteine protease (PCP), a helicase, and an RNA-dependent RNA polymerase (RdRp) are encoded by ORF1. Less characterized domains including Y-domain, the hypervariable region (HVR), and X-domain (Macrodomain) also constitute ORF1 [14]. ORF2 encodes for viral capsid protein while ORF3 which almost entirely overlaps with ORF2 encodes for a small multifunctional ion channel protein involved in viral egress from infected cells [15]. Notably, HEV replication and its life cycle remained poorly understood in terms of ORF1 polyprotein processing, the role of PCP, transcriptional regulation of subgenomic RNA encoding ORF2 and ORF3, and components of HEV replicase complex including essential host factors [3,16,17,18]. Cell culture adapted HEV strains and the development of genome-length and subgenomic infectious complementary DNA (cDNA) clones by reverse genetics [19,20,21,22] have paved the way to understand certain aspects of host–virus interactions during HEV infection [23].

**Figure 2:**
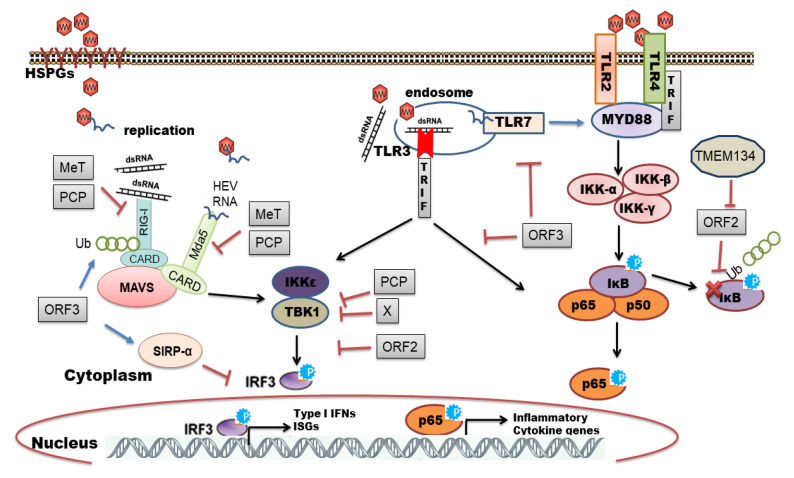
Recognition of Hepatitis E virus (HEV) by host cell pattern recognition receptors (PRRs) and viral strategies to evade antiviral signaling. HEV attaches to host cell heparin sulfate proteoglycans (HSPGs) and enters by clathrin-mediated endocytosis. Release of viral genomic RNA in cytoplasm and RNA replication intermediates are sensed by host cell PRRs like retinoic-inducible gene-I (RIG-I), melanoma differentiation-associated protein 5 (MDA5), and endosomal Toll-like receptor 3 (TLR3). This triggers their downstream signaling cascades, including IRF3 and NF-κB, leading to the production of IFNs and inflammatory cytokines/chemokines. Activation of the inflammatory pathway by replication-defective (UV inactivated) virus through TLR adaptors (MyD88 and TRIF) implicates the plausible role of TLR2 and TLR4 in recognizing HEV capsid structure. On the other hand, HEV has evolved several molecular mechanisms to evade antiviral responses through interplays between viral encoded and host cell proteins. ORF1 encoded proteins PCP and MeT interfere with RLR signaling while X domain, ORF2 and ORF3 inhibit transcriptional activation of IRF3 by reducing its phosphorylation. Conversely, ORF3 enhances IFN signaling by Ubiquitination-mediated stabilization of RIG-I. ORF2 and ORF3 also inhibit NF-κB signaling and ORF2 mediated negative regulation of NF-κB signaling is relieved by interaction and colocalization of cellular protein TMEM134 with ORF2. Abbreviations: IKKε: IκB-kinase-epsilon; TBK1: TANK-binding kinase 1 (TBK1); IRF3: IFN regulatory protein 3; MAVS: mitochondrial antiviral-signaling protein; SIRP-α: signal regulator protein alpha; UV: Ultraviolet; Ub: ubiquitin; TMEM134—Transmembrane Protein 134.

It is proposed that HEV enters host cells through capsid protein interaction with cellular receptors, Heparin Sulfate Proteoglycans (HSPGs), asialoglycoprotein receptor 1/2 (ASGPR1/2), integrin α3 (ITGA3), ATP synthase subunit 5β (ATP5B), glucose-regulated protein 78 (GRP78), and Heat shock cognate protein 70 (HSC70) [16,23]. After entry and uncoating, the ORF1 is translated by host ribosomes into polyprotein containing RdRp. The RdRp uses (+)-sense genomic RNA as a template and makes full-length (−)-ve sense RNA which serves as a template for making genomic (+)-sense RNA and small subgenomic RNA encoding ORF2 and ORF3. Also, multiple steps in virus assembly are poorly understood. ORF2 binding to viral genomic RNA, multimerization of ORF2 as well as an association of ORF3 to ORF2 during assembly and viral egress has been proposed (reviewed in [16,17,18]). During these replication events, viral genomic RNA, double-stranded replication intermediates (dsRNA), and proteins are under strict surveillance by the host cell PRRs to induce innate defense (Figure 2).

The host organism can employ different families of PRRs to sense and immediately respond to a diverse range of pathogens. Individual members of the PRR families can be distinguished by ligand specificity, their cellular localization, and the activation of unique but converging downstream signaling pathways [24,25]. The sensing of PAMPs by PRRs upregulates the transcription of genes involved in inflammatory responses [26]. These genes encode proinflammatory cytokines, type I interferons (IFNs), chemokines, antimicrobial proteins, and proteins involved in the modulation of PRR signaling. In the context of viral infections, Toll-like receptors (TLRs) and retinoic acid-inducible gene I (RIG-I)-like receptors (RLRs) play a pivotal role in recognizing viral PAMPs and eliciting antiviral response [27,28,29]. In this review, we have summarized an interplay between HEV and host cell PRRs along with the mechanisms of subversion of innate immune signaling by HEV.

## 2. Pattern Recognition Receptors (PRRs)

TLRs are present on the cell membrane as well as in endosomes and after recognizing molecular patterns of invading pathogens their signaling is initiated by either of two signaling pathways depending on the signaling adaptor engaged. TLR3 and TLR4 signaling is initiated by using a Toll/IL-1R domain-containing adaptor-inducing IFN- (TRIF) while all TLRs (except TLR3) use Myeloid differentiation primary response 88 (MyD88) [24,25]. Foreign RNA in the cytoplasm is recognized by RLRs; retinoic acid-inducible gene-I (RIG-I, also known as DDX58), melanoma differentiation-associated gene 5 (MDA5, also known as IFIH1), and laboratory of genetics and physiology 2 (LGP2) [27,28,29]. Both RIG-I and MDA5 have tandem N-terminal caspase activation and recruitment domains (CARDs) followed by a DExD/H box RNA helicase domain which has ATPase activity and a C-terminal repressor domain (RD). Unlike RIG-I and Mda5, LGP2 lacks N-terminal CARD domains and contains only the RNA helicase domain. LGP2 is thought to act as a regulator of RLR signaling, a negative regulator of RIG-I signaling, and a positive regulator of MDA5 signaling [29]. RIG-I and MDA5 share a common signaling adaptor: the CARD-containing adaptor protein known as mitochondrial antiviral signaling adaptor (MAVS). After ligand binding, homotypic interactions of CARD-CARD domains between RLRs and MAVS lead to recruitment of other signaling adaptor proteins as well as kinases like IKK complex, TBK1, and IKKε which phosphorylates and activates transcription factors NF-ĸB, IRF3, and IRF7. Upon phosphorylation, IRF3 and IRF7 form homo-and/or heterodimers that translocate into the nucleus, where they transcribe IFN genes. The synthesis of IFN activates JAK/STAT pathway leading to expression of ISGs. IRF3 can also directly interact with interferon stimulatory response element (ISRE) to induce the transcription of certain ISGs [28,29] while NF-ĸB mostly induces inflammatory response [26]. Taken together, TLR and RLR signaling converge on similar pathways leading to synthesis interferons and proinflammatory cytokines to launch an antiviral response [30].

## 3. HEV Sensing by TLRs

In the previous work by one of the authors using ultra-purified HEV (genotype 1) from the patient’s stool, it was observed that HEV elicited an inflammatory response in human lung epithelial cells (A549) by engaging both MyD88 and TRIF adaptors. This study implicated the role of TLR2, TLR3, and TLR4 in recognizing viral capsid and RNA (by TLR3) as soluble ORF2 protein failed to induce response [31]. Small interfering RNA (siRNA) mediated gene silencing of MyD88 and TRIF convincingly showed the dependence of IL-6 and RANTES expression on the MyD88 pathway while IL-8 expression was reduced by the silencing of both signaling adaptors. There was a delayed up-regulation of type 1 interferon genes by replicating virus but the absence of secreted interferons [31]. In continuation with these observations, Li et al. (2015) investigated the molecular mechanisms associated with HEV-induced CXCL-8 (IL-8) transcriptional activation and demonstrated the role of HEV ORF1 in activating CXCL-8 promoter via AP-1 [32]. Clinically, a higher level of TLR3 expression along with anti- and pro-inflammatory cytokines was observed in peripheral blood mononuclear cells (PBMC) of HEV infected patients with acute viral hepatitis compared to patients with acute liver failure [33]. Pregnant women infected with HEV are more prone to develop acute liver failure. To delineate the associated factors, the authors compared the functionality of monocytes and macrophages as well as TLR expression between patients with acute viral hepatitis and acute liver failure. This study revealed impaired monocyte-macrophage functionality and reduced expression of TLR3, TLR7, and downstream signaling molecules in pregnant patients with acute liver failure (ALF), implicating the role of these PRRs in curtailing viral infection [34]. Consistently, Arya and Arankalle (2014) observed reduced expression of TLRs in HEV-infected pregnant women whereas there was a temporal association of TLR4/TLR7/TLR8 at mRNA and protein level in non-pregnant patients with acute hepatitis E [35]. In continuation with this study, TLR4 gene polymorphism (at Asp299Gly and Thr399Ile) was investigated and low TLR4 mediated immune response during HEV infection was suggested to be associated with TLR4 polymorphism [36]. Additionally, a whole transcriptome analysis of PBMC RNA from HEV-infected pregnant women implicated the possible involvement of TLR4 and NOD1 (Nod-like receptor 1) signaling in antiviral defense [37]. Collectively, all aforementioned studies support the indispensable role of TLRs in recognizing HEV-associated molecular patterns and triggering an innate immune response.

## 4. HEV Sensing by RLRs

HEV ORF3 protein is known to enhance poly I:C (double-stranded RNA analog) mediated IFN induction by interaction with RIG-I and extending its half-life through K63-linked ubiquitination [38]. Interestingly, authors noted that ORF3 from genotype 1 and III boosted RIG-I signaling whereas ORF3 from genotype II and IV had a minimal effect [38]. It will be interesting to explore genotype-specific virulent factors in the host tropism of HEV which remains an open question. Using the HEV genotype 1 replicon system, we could show altered replication efficiency of HEV in different human hepatoma cell lines (Huh7, Huh7.5, and HepG2/C3A) due to cell-type-specific innate immune responses [39]. We identified the role of RIG-I and TLR3 in sensing HEV RNA and activation of downstream interferon regulatory factor 3 (IRF3) mediated antiviral responses. Inhibition of this signaling cascade downstream of PRRs by pharmacological inhibitor BX795 significantly improved HEV replication efficiency [39]. Furthermore, overexpression of interferon regulatory factor 1 (IRF1) effectively inhibited HEV replication through the JAK-STAT signaling cascade independent of interferons. The authors revealed transcriptional activation of STAT1 promoter by IRF1 resulted in the elevation of intracellular STAT1 levels and transcription of ISGs [40]. The same research group in 2017 identified RIG-1 as a key anti-HEV ISG in cell culture models by overexpression studies [41]. The pharmacological activation of RIG-I by its natural ligand (5′-pppRNA) was identified to combat HEV infection which was independent of IRF3/IRF7 activation and IFN induction [41]. The authors proposed two distinct mechanisms of RIG-I mediated ISG induction viz. JAK-STAT dependent and independent [41]. The integrity of JAK-STAT signaling in antiviral defense against HEV was confirmed by immunohistochemical staining of transcriptionally active phospho-STAT1 (Y701) in liver biopsy samples of acute or chronic HEV [42]. Single-stranded HEV genomic RNA potently induced innate immune response in cell lines as well as in primary three-dimensional liver organoid cultures [42]. Moreover, HEV genotype 3 specific stable replicon system in human (Huh-7-S10-3) or hamster (BHK-21) origin cells also revealed the role of RIG-I and IRF3 in antiviral defense during HEV replication [43].

The second RLR, Mda5 was seen to be upregulated at 48 h post-infection in epithelial cells (A549) during HEV infection in our TaqMan low-density array (TLDA) based transcriptome analysis study [31]. In hepatoma cell lines HepG2/C3A and Huh7:S10-3 transfected with HEV subgenomic RNA, Mda5 expression was higher after 24 h post-transfection and it was associated with viral RNA replication as Mda5 expression was comparatively less in cells transfected with replication-deficient RNA [39]. Recently, Li et al. (2020) demonstrated the role of Mda5 in inhibiting HEV replication in both HEV infectious and subgenomic replicon models [44]. The overexpression of Mda5 mediated its antiviral action by inducing a wide range of antiviral ISGs independent of interferon production through partial activation of JAK-STAT signaling [44].

The functional knockdown of RLR signaling mediators; RIG-I, Mda5, and MAVS in liver cells revealed the reduction of HEV induced type III IFNs which implicated the role of both RLRs in sensing HEV RNA [45]. Moreover, transcriptome analysis of primary human hepatocytes infected with genotype 3 HEV revealed expression of PRRs; RIG-I, Mda5, TLR3, and downstream signaling molecules MyD88 and MAVS, supporting the physiological role of innate immune signaling during HEV infection [46]. Both RIG-I and Mda5 are known to recognize specific molecular patterns in foreign RNA. 5′-ppp RNA [47], capped dsRNA, as well as size and composition of the RNA ligand are demonstrated as some important features specifically identified by RIG-I and Mda5 [48,49]. Homopolyuridine or homopolyriboadenine motifs present in the genomes of RNA viruses are the chief features of RIG-I recognition in human and murine cells [50]. In this regard, Sooryanarain et al. (2020) attempted to identify HEV RNA-associated molecular patterns in eliciting RIG-I mediated antiviral response [51]. The authors identified the U-rich region in the 3′ untranslated region (UTR) of the HEV genome as a potent RIG-I agonist.

## 5. HEV Infection-Mediated Interferons (IFNs)

IFNs are grouped into type I, type II, and type III IFNs based on their amino acid composition. Type I IFNs (often abbreviated as IFN-α/β), Type II IFNs (IFN-γ), and Type III IFNs comprise IFNλ1 (IL-29), -λ2 (IL-28A), -λ3 (IL-28B) and a newly discovered subtype IFNλ4. These cytokines use the same pathway of activation as that of IFN-α/β in response to direct viral infection by engaging distinct receptor complexes [27,52]. Exogenous treatment by all three types of IFNs exhibited antiviral activity against HEV in subgenomic and full-length genome cell culture models [53]. IFN-α has been identified as a potent antiviral factor inhibiting HEV replication [39,53,54]. Genotype 3 HEV infection in HepG2 cells and primary human hepatocytes induced type III IFNs and virus replication persisted in presence of type III IFNs. Interestingly, among type III IFNs, the expression of IFN-λ1 and IFN-λ2/3 was found to be upregulated while IFN-λ4 remained unchanged following HEV infection [45]. This distinct regulation of IFN-λ subtypes during HEV pathogenesis remains obscured. IFN-λ4 is a newly discovered subtype present upstream of the IFN-λ3 gene which was reported to be associated with treatment-induced clearance of Hepatitis C virus (HCV) [55]. Similarly, an association of favorable IFN-λ4 polymorphism and treatment with high-flux hemodialysis were reported to be determinants of anti-HEV IgG positivity (spontaneous HEV resolution) among hemodialysis patients exposed to HEV [56]. Stem cell-derived hepatocyte-like cells were shown to be permissive for infection by all four HEV genotypes (genotype 1 to 4) [57]. Cell culture adapted genotype 3 HEV infection elicited induction of IFN-β, -λ1, and -λ3 expression at mRNA level whereas IFN-α expression remained undetectable in these hepatocyte-like cells. However, at protein level only type III IFN levels were higher at 7- and 9-days post-infection while type I IFNs remained undetected in cell culture supernatants [57]. Consistent with these in vitro results, the pig model system of genotype 3 HEV infection also showed predominant induction of type III IFNs in liver tissues [51]. This HEV-induced IFN induction was observed to be cell type-dependent manner as there was predominantly type III IFN response in human and pig hepatocytes, while type I IFN induction was noted in HEV infected pig enterocytes [51]. HEV can replicate in primary human intestinal cells and infection of these cells with clinically isolated (stool-derived) genotype 1 and -3 HEV profoundly induced more type III IFNs than type I IFNs [58]. Recently, high levels of IFN-λ3 were detected in serum samples of acute hepatitis E patients during the early phase of the infection which was also correlated with viral RNA in serum. Furthermore, the antiviral activity of IFN-λ3 in a dose-dependent manner was verified in the cell culture model [59]. Altogether, the majority of the studies reveal the role of type III IFNs followed by type I IFNs in controlling HEV infection.

## 6. Induction of Interferon Stimulated Genes (ISGs) by HEV

**Figure 3:**
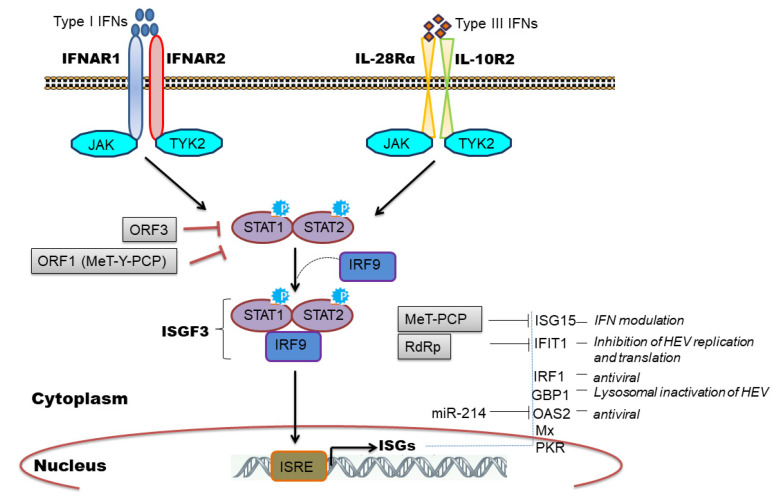
A model depicting induction of Type I and type III IFNS by engaging IFNAR1/IFNAR2 and IL-28Rα/IL-10R2 receptors, respectively and strategies evolved by HEV to evade host innate response. Type I and type III IFNs binds to their respective receptors which results in phosphorylation of STAT1 and STAT2. Phosphorylated STAT1 and STAT2 forms complex with IRF9 (ISGF3) which is translocated in nucleus to transcribe ISGs from ISRE promoter element. ISGs have different antiviral mechanisms while HEV employs different strategies to evade IFN response.

The IFN signaling cascade begins with the engagement of heterodimeric receptors (IFNAR1/IFNAR2 or IL-28Rα/IL-10R2) by IFNs which activates Janus kinases (JAK), Jak1, and Tyk2, which in turn phosphorylate STAT1 (signal transducers and activators of transcription1) and STAT2 transcription factors. Subsequently, STAT1 and -2 heterodimerize and interact with IRF9 to form the heterotrimeric cytoplasmic complex, called ISGF3. This complex translocate into the nucleus, binds to genes containing IFN-stimulated response elements (ISREs), and induces expression of ISGs which are major antiviral effectors [27,52] (Figure 3).

We and others have observed induction of ISGs during HEV replication [31,39,41,42,43,45,46,60,61,62] and clinically ISG induction has been related to HEV persistence in chronic HEV patients [63]. Furthermore, the constitutive expression of ISGs was mediated by unphosphorylated ISGF3 complex to inhibit HEV replication [64] and the knockdown of individual ISGF3 components; IRF9, STAT1, and STAT2 reversed the antiviral effect of ISGs and increased viral replication [54,64]. Few studies have attempted to characterize the antiviral role of individual ISGs in regulating HEV infection. To illustrate, ectopic expression of 25 ISGs in genotype 3 HEV cell culture model identified RIG-I, Mda5, and IRF1 as major ISGs inhibiting HEV replication [41]. IRF1 is another important ISG that was shown to inhibit HEV replication by the induction of antiviral ISGs through the JAK-STAT pathway but independent of IFN induction [40]. Interestingly, targeting the nucleotide synthesis pathway was linked with ISG induction and anti-HEV effects, independent of JAK-STAT signaling [65]. However, the mechanistic insights related to nucleotide biosynthesis pathway and cellular immune responses remain elusive. ISG15 is a ubiquitin-like modifier protein induced by IFNs and gets conjugated onto different target proteins (ISGylation) or secreted as a cytokine to regulate diverse functions [66]. An increase in ISG15 mRNA levels in liver biopsies of HEV infected chimpanzee [60], chronic HEV patients [63], and cell culture models of HEV infection [31,39] implicated its role during HEV replication. In this context, an immunomodulatory role of ISG15 was proposed [67,68] wherein ISG15 expression was induced at mRNA and protein level in HEV infected cells as well as in pig livers [68]. Knockdown of ISG15 enhanced expression of type I IFNs [67] and IFN mediated antiviral response against HEV by inducing expression of other ISGs like PKR and OAS1 [68]. ISG15 is also induced by unphosphorylated ISGF3 complex [64] and might be causing IFN unresponsiveness in HEV infected cells by maintaining a sustained expression of ubiquitin-specific protease 18 (USP18), a negative regulator of IFN signaling [69]. IFIT1 (Interferon-induced protein with tetratricopeptide repeat 1) is an IFN induced antiviral protein known to recognize viral RNAs without the 2′-OH-methylated cap (Cap0) or with a free 5′-ppp RNA and thereby regulating viral RNA translation [70]. We observed an upregulation of this gene (ISG56) in genotype 1 HEV infected or replicon transfected cells [31,39] while genotype 3 HEV also induced IFIT1 expression in HepG2/C3A cells [45]. Recently, Pingale et al. (2019) demonstrated the functional association of IFIT1 with HEV replication [71]. The authors showed that overexpression of IFIT1 inhibited HEV replication by interacting with HEV RNA and affecting its translation [71]. Transcriptionally GBP1 (guanylate-binding protein 1) was found to be elevated at 48-h post-infection in HEV infected cells (A549) compared to UV inactivated HEV infection as well as in replicon transfected hepatocyte cell lines [31,39]. Very recently, the role of this antiviral factor against HEV was established. Authors revealed that GBP1 exerted its antiviral role by the homodimerization and independent of GTPase activity wherein GBP1 targeted virus to the lysosomal compartment to inactivate it [72].

Few in vivo studies have documented the use of animal models to understand HEV pathogenesis [73]. Yu et al. (2010) compared Microarray-based transcriptome profiling between HCV- and HEV-infected chimpanzees. Both the viruses elicited different magnitude of immune response whilst in HCV it was more robust compared to HEV infection. However, there was a significant increase in ISG expression in the livers of HEV-infected chimpanzees [60]. Genotype specific difference in host immune response of rhesus macaques was also noted when they were infected with genotype 1 and 3 HEV [61]. Notably, the gene expressions varied in different phases of infection and during early viremia, hepatic immune-response related genes were down-regulated in genotype 1 infected animals compared to genotype 3 infection [61]. Homologous HEV genotype 1 re-infection in seroconverted rhesus macaques showed upregulation of mitogen-activated protein kinase (MAPK) signaling suggesting antiviral oxidative stress response [74]. The human liver chimeric mouse model developed for HEV genotype 1 and 3 revealed the induction of ISGs in genotype 1 infected liver tissue and the induction of innate immune response was more pronounced at 9 days post-infection compared to 4-week post-infection [62]. Pigs are also considered as a model system for the propagation of genotype 3 and 4 HEV infection [75,76]. Recently, a predominant type III IFN response was observed in liver tissues of HEV genotype 3 infected pigs [51].

## 7. Induction of Inflammatory Response by HEV

Hepatitis E is an inflammation-mediated liver disease and there is some evidence on the inflammatory response in HEV infection. A comparative determination of Th1 and Th2 cytokines from acute viral hepatitis (AVH) and fulminant hepatic failure (FHF) patients revealed a remarkable difference in cytokine profile. IL-12 (Th1) cytokine levels were elevated in AVH patients compared to controls while both Th1 (IGN-γ, IL-2, and TNF-α) and Th2 (IL-10) cytokines were elevated in FHF patients compared to AVH category [77]. Similarly, the isolation and stimulation of PBMCs from acute hepatitis E and recovered patients by recombinant ORF2 protein elicited elevation of IL-1β, TNF-α, and IL-10 compared to healthy counterparts while cytokines IL-1β and TNF-α were specifically elevated in recovered patients [78]. This study also established a positive correlation of regulatory T-cell induction and an increased IL-10 level in ORF2 stimulated PBMCs implicating the involvement of immunosuppressive response during HEV infection. Functional relevance of regulatory T-cells was established in acute hepatitis E patients and recovered individuals by determining the expression of Foxp3, IL-10, and TGF-β. The suppressive activity of Treg cells was higher in patients with acute hepatitis E compared to recovered individuals [79]. Moreover, higher level of IL-1α and sIL-2Rα from blood samples of acute hepatitis E patients implicated their role in HEV pathogenesis as inflammatory markers [80]. High levels of TLR3 and anti-and pro-inflammatory cytokines; IFNγ, TNF-α, IL10, and TGF-β in acute viral hepatitis patients compared to acute liver failure patients were found to play an important role in HEV disease pathogenesis [33]. As evident from in vitro study by one of the authors, induction of inflammatory cytokines/chemokines including IL-6, IL-8, TNF-α, and RANTES was observed within 12 h of infection in genotype 1 HEV infected lung epithelial cells [31]. This response was dependent on TLR adaptor proteins (TRIF and MyD88), elicited by native and UV inactivated virus which suggested recognition of viral capsid as PAMP by host cell PRRs. The antiviral activity of TNF-α was demonstrated by treating HEV genotype 1 replicon transfected Huh7 cells wherein TNF-α displayed anti-HEV activity through induction of ISGs [81]. HEV infection in primary enterocytes elicited an inflammatory response of different magnitude by different strains. All HEV strains including Kernow-p6 strain and clinical HEV isolates (genotype 1 and 3 from stools of infected patients) elicited IL-1α, CXCL5, and CXCL8 (IL-8) secretion while Kernow-p6 strain-induced production of IL-6, CXCL10, and CCL5 [58]. To understand the genotype-specific pathogenicity of HEV during pregnancy, organ cultures from the maternal decidua and fetal placenta were infected with genotype 1 and 3 HEV. There was prominent induction of IL-6, CCL3, and CCL4 in genotype 1 infected decidua and placenta compared to genotype 3 while expression of TNF-α, IL-15, IL-18, and CCL-5 was barely detected regardless of infection [82]. The HEV-mediated inflammatory response was also confirmed in animal models. Swine HEV isolate was used to infect the Mongolian gerbil model which induced high levels of pro-inflammatory cytokines TNFα, IL-1β, and IBA1 (ionized calcium-binding adapter molecule 1) in brain tissues. The authors proposed this response in the perspective of HEV-induced mitochondrial apoptosis and brain injury [83]. Recently, a higher inflammatory response was found to be associated with advanced liver disease in HEV seropositive obese patients [84].

## 8. Viral Evasion and Subversion of Innate Immune Response

The lack of extracellular type I IFNs in HEV infected cell culture supernatants [31], as well as the absence of IFN gene expression in HEV infected rhesus macaques [61], implicates the ability of HEV to modulate IFN response. Also, from experimental evidence and pegylated IFN-α treated chronic hepatitis E patients, it is proposed that HEV is less sensitive to exogenous IFN treatment compared to HCV and might be inducing potent anti-interferon mechanisms [53,54,81,85]. Despite elevated levels of type III IFNs and ISGs, HEV persisted in liver cells and rendered infected cells refractory to high doses of IFNs suggesting unknown immune evasion mechanisms by HEV [45,57]. It is intriguing to know how HEV manages to replicate in presence of antiviral defense. Several studies have reported the role of viral structural and non-structural proteins in evading innate immune defense (Figure 2 and Figure 3). There is an interplay between RLRs and non-structural proteins of HEV. To illustrate, HEV replication in hepatoma cells inhibited poly I:C induced IFN-β signaling and ORF1 domains; ‘X’ and ‘PCP’ were identified as putative IFN antagonists [86]. X-domain inhibited poly I:C mediated phosphorylation of IRF3 while deubiquitination of RIG-I and TBK-1 by PCP inhibited downstream PRR signaling cascade [86]. Moreover, amino-terminal ORF1 region of genotype 3 HEV containing Methyltransferase, Y- and PCP domains inhibited type-I IFN induced ISRE promoter activity and expression of ISGs by interfering with STAT1 phosphorylation and nuclear translocation [87]. Ubiquitination-mediated regulation of innate immune response proteins is well established [88] and a previous report has suggested the deubiquitylation activity of HEV PCP in a cell-free system implicating an essential role of PCP in curtailing innate immune response [89]. HEV PCP and Methyltransferase both inhibited Mda5 and RIG-I mediated IFN induction through NF-κB and IRF3 arms [90,91,92]. However, these studies were based on an exogenous expression of virally encoded genes and the underlying molecular mechanisms of RLR regulation by methyltransferase and PCP remain obscure during the course of HEV replication. Using a Yeast two-hybrid system, X-protein (Macrodomain) was reported to interact with human acute-phase protein; ferritin and this interaction were confirmed in Huh7 S10-3 cells wherein macrodomain inhibited ferritin secretion from cells [93]. Thus, by sequestering ferritin, the macrodomain may restrain cellular innate immune response but this association needs further confirmation in a suitable HEV infection system. HEV polymerase (RdRp) was also proposed to overcome the IFIT1-mediated antiviral immune response [71]. It was found that IFIT1 binds to HEV RNA and inhibits its translation which was relieved by RdRp mediated sequestration of IFIT1 [71]. These observations provide compelling insights into HEV ORF1 mediated evasion of host antiviral response.

Proteins encoded by other ORFs; ORF2 and ORF3 are also observed to modulate antiviral responses (Figure 2). ORF2 was reported to interfere with host cell apoptotic signaling to favor the virus replication cycle [94]. Surjit et al. (2012) demonstrated ORF2 mediated the inhibition of NF-κB transcriptional activity by stabilizing cellular I kappa B alpha (IκBα) through disruption of its ubiquitination [95]. However, an interaction of host transmembrane protein 134 (TMEM134) with ORF2 was implicated in negative regulation of ORF2 mediated inhibition of NF-κB signaling [96]. This is in accord with our findings of predominant NF-κB promoter activity and inflammatory response in HEV-infected cells [31]. Both genotype 1 and 3 encoded ORF2 could inhibit Poly I:C and Sendai virus induced IFN induction by blocking IRF3 phosphorylation [97]. Authors revealed that ORF2 interacts with multiprotein signaling complex MAVS-TBK1-IRF3 to inhibit IRF3 phosphorylation and its release from the complex [97]. Hingane et al. (2020) confirmed that ORF2 inhibits both TLR and RLR mediated interferon response by lowering the nuclear localization of IRF3 [98].

ORF3 identified as a phosphoprotein of 13 kDa is a multifunctional protein that has been proposed to create a favorable environment for virus replication [99]. The localization of ORF3 in early and recycling endosomes delays Epidermal Growth Factor Receptor (EGFR) trafficking, thereby lowering STAT3 translocation efficiency from the cytoplasm to the nucleus. This resulted in the downregulation of STAT3-responsive acute-phase response genes [100]. Dong et al. (2012) saw an inhibition of IFN-α signaling by genotype 3 HEV by preventing the phosphorylation of STAT1 [101]. Furthermore, the authors showed association of ORF3 with STAT1 as well as ORF3 expression alone was sufficient to inhibit STAT1 mediated ISG induction. Conversely, Genotype 1 and 3 ORF3 was found to enhance IFN-β promoter activity by polyubiquitination mediated stabilization of RIG-1 and this effect was genotype-specific as genotype 2 and 4 ORF3 had a minimal effect on RIG-I activation [38]. Similarly, genotype 3 encoded ORF3 when overexpressed in HEK293T cells elicited Poly I:C induced IFN-β promoter activity [91]. Genotype 3 HEV infection in HepG2/C3A cells enhanced production of type I IFNs and ISG15 expression through ORF3 protein [67] and increased ISG15 level modulated IFN induction. The authors proposed the role of ORF3 in regulating cellular antiviral response through ISG15 as the virus lacking the ORF3 coding sequence elicited less ISG15 [67]. Genotype 4 HEV ORF3 activated SIRP-α (Signal regulator protein α) and thereby down-regulated IRF3 phosphorylation which ultimately inhibited IFN-β expression [102]. These contradictory observations need further investigation to determine the role of ORF3 in modulating antiviral response at different stages of replication.

HEV ORF3 has been shown to interfere with TLR signaling. Genotype 1 HEV ORF3 inhibited TNF-α mediated NF-κB signaling in A549 cells [103]. In an attempt to delineate the underlying mechanism same research group demonstrated reduced expression of tumor necrosis factor receptor 1-associated death domain protein (TRADD) and receptor-interacting protein kinase 1 (RIP1) in Poly I:C stimulated and ORF3 expressing cells which resulted in suppression of TLR3 mediated NF-κB signaling [104]. In line with this, ORF3 also inhibited TLR3 and TLR7 expression, thereby impairing the generation of endogenous type I IFNs. There was a significant downregulation of multiple signaling pathways, including NF-κB, JAK/STAT, and JNK/MAPK when stimulated by their respective agonists TNFα, IFNγ, and Anisomycin in ORF3 expressing cells [105]. HEV ORF3 attenuated lipopolysaccharide (LPS) -induced cytokine and chemotactic factor production in differentiated monocyte-derived cells (PMA-THP1) by inhibiting various PRR- (TLR4, TRAF6, and NOD2) mediated NF-κB signaling [106]. LPS in the serum from patients with HEV-related acute liver failure was found to induce liver cell apoptosis [107]. HEV ORF3-mediated regulation of LPS-induced signaling [106] might be attenuating the endotoxemia in the liver during the natural course of infection as proposed for HCV NS5A protein [108]. HEV ORF3 mediated the impairment of phagocytosis by downregulation of phagocytic receptors CD14 and CD64 through the inhibition of JAK/STAT signaling is also demonstrated [109]. Overall, these findings implicate the role of ORF3 in curtailing cellular antiviral response and favoring virus replication.

## 9. Noncoding RNA: LncRNA and MiRNA

Noncoding RNAs (ncRNAs) are emerging as an essential biomolecule in controlling different cellular functions and cell survival. ncRNAs can play a pro-viral role by regulating cellular antiviral response or their regulation can help host cells to counteract viral replication [110]. For example, IFN-α induced lncISG15 and lncBST2 (also called lncRNA-BISPR) are reported as positive regulators of ISGs ISG15 and BST2 [111]. BST2/Tetherin is an antiviral ISG and known to inhibit virus budding [111]. Both BST2 and lncRNA-BISPR were seen to be upregulated in HEV replicon transfected Huh7 cells and there was a co-localization of BST-2 and ORF2 protein. By generating BISPR gene knockout cell line, the authors observed ~8-fold increase in viral egress. These results proposed a role of the BISPR/BST2 axis in regulating HEV egress from the infected cells [112].

MicroRNAs (miRNAs) are highly conserved small non-coding RNAs of about 21-to-23-nt long in their mature forms and are mainly involved in posttranscriptional gene regulation [113]. The role of miRNAs in regulating multiple cellular processes including cellular inflammatory and innate immune responses [114] during HEV replication are underexplored to date. HEV genotype 1–4 sequences harbors miR-122 target site and overexpression of miR-122 reported to enhance genotype 1 and 3 HEV replication in hepatocytes [115]. However, increased miR-122 levels did not modulate cellular antiviral response to enhance HEV replication. The miR-214 was elevated during HEV replication, and its binding site is seen to be conserved among all HEV genotypes in the ORF1 region. miR-214 contributed to positive regulation of HEV replication by increasing pro-viral intracellular thrombin level by targeting its negative regulator protein C (PROC). miR-214 also targeted the host antiviral factor; 2′–5′-oligoadenylate synthetase (OAS2) which is an ISG known to degrade viral RNA through RNase L-dependent or -independent pathways [116]. Thus, exploring the role of cellular non-coding RNAs in modulating host antiviral response during HEV replication will help to understand complex pathological outcomes during HEV infection.

## 10. Antiviral Treatment

There is no specific antiviral treatment against HEV and only a licensed HEV vaccine named Hecolin (Xiamen Innovax Biotech, Xiamen, Fujian, China) is not available outside China [117]. The antiviral drugs Ribavirin and pegylated IFN alpha are the currently used treatment modalities to treat acute and chronic HEV infections with their own limitations and substantial anti-HEV activity. Ribavirin monotherapy has shown HEV clearance in 95% of patients from a cohort of solid organ transplant recipients with chronic HEV infection [118]. However, both Ribavirin and pegylated IFN alpha are contraindicated in pregnancy [119]. Also, there are differences in PRR expression and production of inflammatory cytokines in pregnant women compared with non-pregnant counterparts [35,37]. This dysregulation may have poor prognosis in HEV infected pregnant women. The emergence of less sensitive or resistant virus isolates during ribavirin treatment [85,120,121] and an increased risk of transplant rejection after pegylated IFN alpha therapy limit their use. Moreover, from an in vitro study using HEV subgenomic replicon system, Todt et al. (2016) observed no additive antiviral effect in combination of IFN alpha subtypes with ribavirin [53]. In addition, less sensitivity of HEV to IFNs [45,53,54] in in vitro studies needs careful assessment in considering IFNs as an alternative therapeutic modality against HEV. Thus, few challenges remain in treating pregnant women and patients who fail on ribavirin therapy, especially immunocompromised solid organ transplant recipients. There is an immediate need for extensive research in the field of designing novel- anti-HEV treatment strategies.

## 11. Conclusions and Future Perspectives

The development of infectious complementary DNA (cDNA) clones, subgenomic replicons, and HEV-adapted cell culture models [16] have substantially evaluated the role of host cell encoded PRRs in eliciting antiviral response against HEV. In turn, HEV manages to replicate in presence of antiviral defense by modulating key signaling pathways by virally encoded proteins as revealed in the aforementioned studies. Although these studies have attempted to reveal underlying molecular mechanisms during HEV replication and pathogenesis, certain knowledge gaps need further attention. The majority of the observations are either from overexpression of viral proteins in different cell culture systems or using recombinant HEV strains and hence clinical relevance during the natural course of HEV infection remains obscure. It will be interesting to reveal genotype-specific innate immune response elicited by HEV since genotypes 1 and 2 usually lead to self-limited infections and genotypes 3 and 4 can persist in immunocompromised patients despite the induction of antiviral response. Genotype-specific differential disease outcomes need further investigation. Importantly, genotype 1 HEV seems to be more virulent in developing countries during pregnancy causing ~30% mortality especially in the third trimester and the underlined mechanisms are still in infancy. Whether genotype-specific viral proteins such as PCP and ORF3 modulate antiviral response differently remains elusive. The presence of additional ORF4 exclusively in genotype 1 HEV and not encoded by other genotypes makes it clinically more virulent will be interesting to know. ORF4 was reported to enhance genotype 1 HEV replication by increasing RdRp activity in response to endoplasmic reticular stress. Most importantly, the identification of HEV variants containing proteasome resistant ORF4 isolated from fulminant hepatic failure (FHF) and acute hepatitis patients increased viral replication suggesting poor clinical outcomes [12]. Further studies are essential to understand if ORF4 also subverts antiviral immune response under stress conditions and helps genotype 1 HEV to establish infection. The complex interplay between HEV and host cell antiviral responses needs in-depth understanding considering emerging genotypes, zoonotic transmission, and hepatic and extra-hepatic manifestations.

## Figures and Tables

**Table 1:** Classification of the *Hepeviridae* family.

Genus	Species	Genotype	Natural Host
*Orthohepevirus*	*Orthohepevirus A*	1	Human
		2	Human
		3	Human, Pig, Rabbit
		4	Human, Pig
		5	wild boar
		6	wild boar
		7	Dromedary camel, human
		8	Bactrian camel
	*Orthohepevirus B*		chicken
	*Orthohepevirus C*	C1	rat, greater bandicoot rat, Asian musk shrew
		C2	ferret, mink
	*Orthohepevirus D*		bat
*Piscihepevirus*	*Piscihepevirus A*		cutthroat trout
